# Breast-conserving surgery is contraindicated for recurrent giant multifocal phyllodes tumours of breast

**DOI:** 10.1186/1477-7819-12-213

**Published:** 2014-07-15

**Authors:** Elroy P Weledji, George Enow-Orock, Marcelin N Ngowe, Leopold Aminde

**Affiliations:** 1 Department of Surgery, Faculty of Health Sciences, University of Buea, P.O. Box 126, Limbe, S.W. Region, Cameroon; 2 Department of Pathology, Regional Hospital Buea, Limbe, Cameroon

**Keywords:** Breast-conserving, Giant cytosarcoma phyllodes, Simple mastectomy

## Abstract

**Background:**

The controversy between breast conserving surgery and simple mastectomy for phyllodes tumours of the breast remains because of the unpredictable nature of the disease. Although some benign tumours may show an unusually aggressive behaviour, modified radical surgery for phyllodes tumours offers no survival advantage, and recently more conservative surgical approaches have been deployed.

**Case presentation:**

A 30-year-old woman with a giant multifocal tumour of the breast underwent breast-conserving surgery that made use of the well- circumscribed feature of the tumour. The case demonstrates the safety, and cosmetic benefit of the breast-conserving approach for multifocal phyllodes tumours except for the high recurrence rate.

**Conclusions:**

Large size, multifocality, and borderline or malignant histology are contraindications for breast-conserving surgery.

## Background

Phyllodes tumours of the breast are rare and account for less than 1% of breast tumours [1]. Malignant transformation of a phyllodes tumour is a rare form of breast cancer accounting for just 0.5% of all breast cancers [1,2]. Phyllodes tumours are fibroepithelial tumours composed of a benign epithelial component and a more numerous cellular spindle cell stroma forming characteristic broad -“leaf-like”- (*phullon)* papillae inserted into cleft-like epithelial spaces. The hypercellular stroma is the neoplastic component [3]. The median size of phyllodes tumour is around 4 cm. Twenty percent of tumours grow larger than 10 cm- the arbitrary cut-off point for designation as a giant tumour [1-3]. Studies have shown no difference between breast- conserving surgery versus mastectomy in terms of metastases free-survival or overall survival despite the higher incidence of local recurrence that comes with breast-conserving surgery [4,5]. Arguments for over- and under- treating these patients remain due to the difficulty of forming a clear diagnostic and therapeutic pathway in this highly variable disease. Wide excision with a clear surgical margin is the preferred therapy for phyllodes tumour. Re-excision is recommended in cases with a positive surgical margin and stromal overgrowth and malignancy [4]. The giant phyllodes presents with several unique management problems. A simple mastectomy is performed for giant phyllodes tumours (>10 cm), those that are multifocal, in cases of recurrence or in phyllodes of ‘borderline’ histology [5]. Breast-conserving surgery is traditionally avoided in large multifocal phyllodes because of the risk of inadequate local excision and associated high local recurrence [5-8]. We report a case of a recurrent, rapidly growing but clinically benign giant multifocal phyllodes tumour of the breast that was treated by breast-conserving surgery for better cosmesis.

## Case presentation

**Figure 1:**
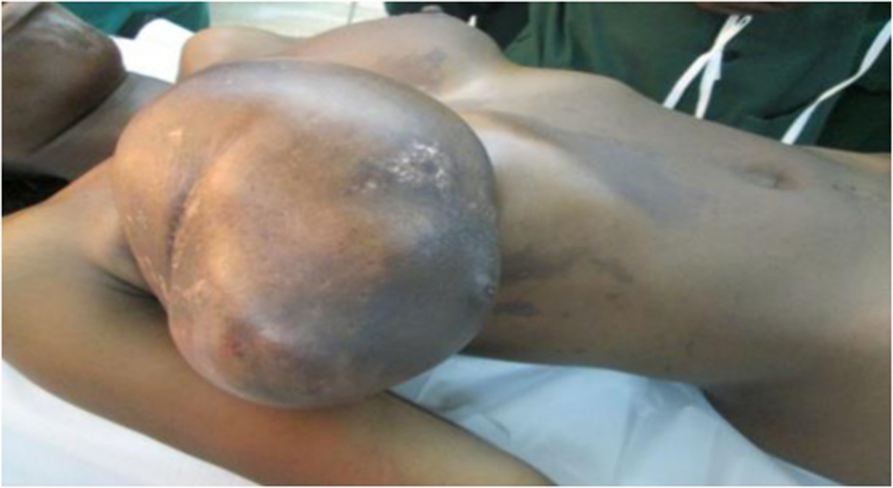
Giant phyllodes tumour of breast.

**Figure 2:**
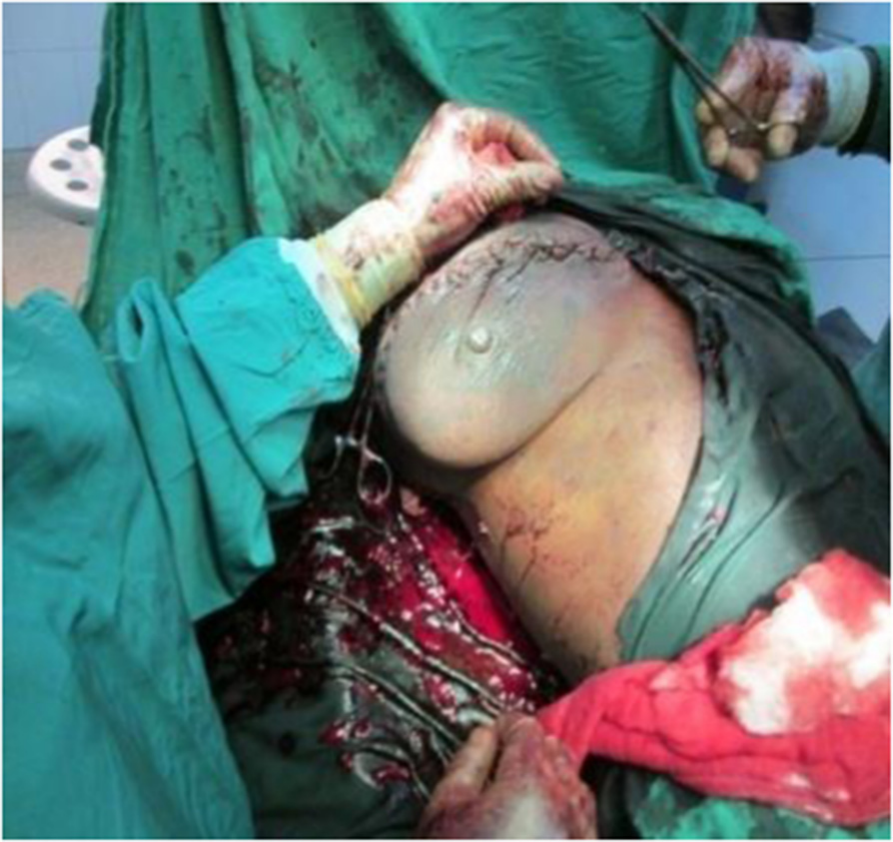
Breast-conserving surgery for phyllodes tumour of right breast. (Note redundant skin).

**Figure 3:**
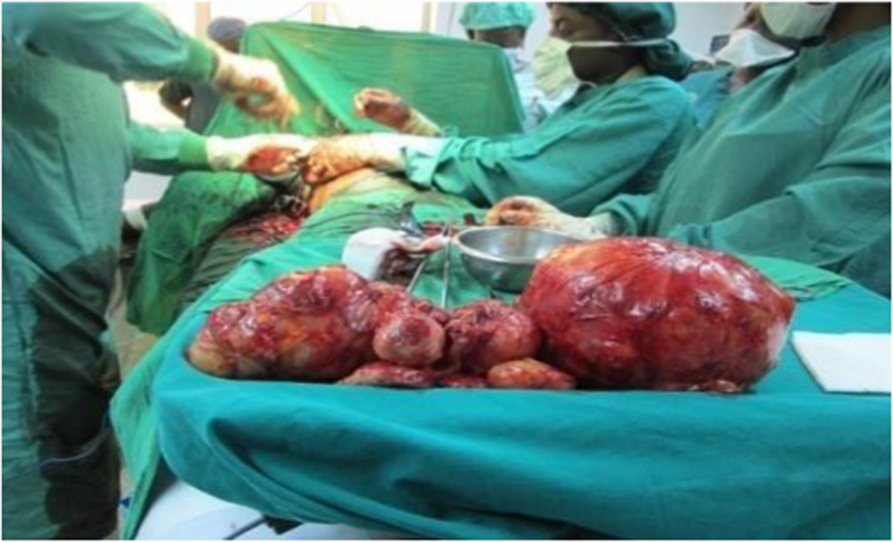
Operative Specimens (Phyllodes tumours).

**Figure 4:**
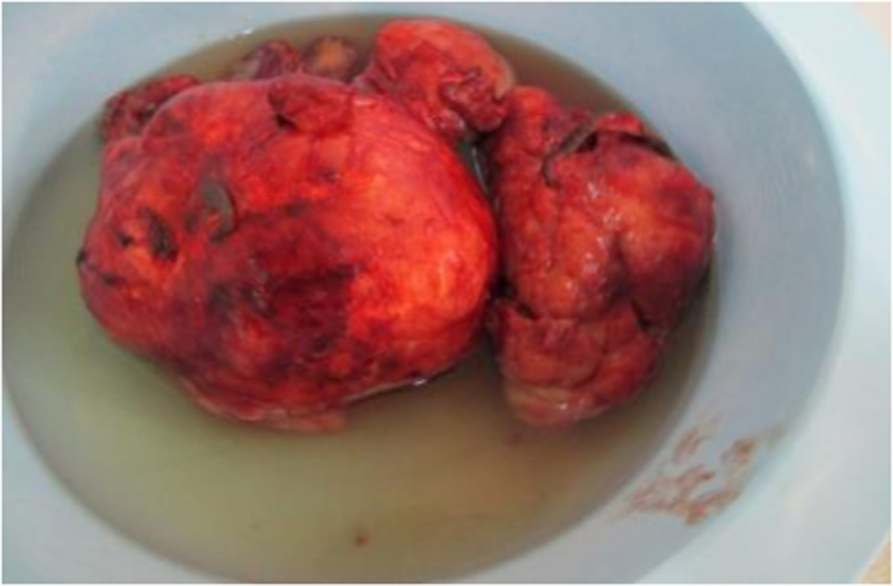
Fleshy appearance with cystic and necrotic areas.

A 30- year- old lactating mother was admitted electively for investigation of a recurrent rapidly growing mass in her right breast over a period of 11 months. The mass was initially noticed as a small painless lump in the upper, outer quadrant of her breast when she was five months pregnant. Results of a fine needle aspiration cytology suggested a fibroadenoma. An ultrasound examination revealed heterogeneous, hypoechogenic masses with cystic portions of 86 mm and 62 mm in diameter and two axillary lymph nodes of 17 mm and 15 mm but a definitive diagnosis could not be made. Her left breast was entirely unaffected. She breast- fed her current four- month- old baby from both breasts. Two years previously, she underwent the excision of a heterogeneous right-breast mass but no precise diagnosis was made as the operative specimen was not histologically examined.On examination she appeared clinically well with no pallor or weight loss. Both breasts were lactating. Her left breast was normal on palpation. Her right breast was extremely large and heavy with palpable large focal lumps. The major lump was greater than 10 cm in total diameter (Figure 1). They appeared encapsulated and firm in consistency. The masses were attached to adjacent breast tissue but not to underlying muscle nor to overlying skin. There was no skin tethering or nipple retraction but a transverse scar in the upper outer quadrant. The whole breast mass moved freely over the pectoralis major and there were no palpable axillary or supraclavicular lymphadenopathy. Chest and abdominal examination were unremarkable. Her haemoglobin level and a chest X-ray were normal.Our patient gave consent to a wide local excision of the tumour mass and not to mastectomy, despite being informed of the risk of local recurrence. An elliptical skin incision was made at the upper part of her breast over the biggest mass and included the previous scar. A subcutaneous flap was developed superiorly and inferiorly. A cleavage plane of the biggest tumour (>10 cm in diameter) in the upper inner quadrant was identified and the tumour excised. Using the same incision, multiple wide tumourectomies (enucleations) were performed. Two further tumours in the outer upper and lower quadrants were excised measuring about 8 cm in diameter. Six further smaller tumours within the breast tissue were removed (Figure 2). The tumours were identified on palpation by their firmer consistency and circumscribed margins as compared to normal breast tissue or the other breast. There was no infiltration to the underlying pectoralis major muscle. There were no palpable axillary lymph nodes intraoperatively and axillary dissection was not performed.Meticulous haemostasis was done with electrocautery and suture ligation. The estimated blood loss was about 300 ml. The normal breast tissue was approximated and the wound closed in layers. The skin was closed with interrupted sutures. No drain was inserted. The residual right breast volume was almost the same as the left (Figure 3). The total operation time was 2 hours 30 minutes. She received prophylactic antibiotics and analgesia. Her post operative haemoglobin level was 8 g/dl for which she received iron supplements. She also received prophylactic antibiotics and analgesia. She was discharged on the fifth post operative day with a three-monthly follow-up.The histopathological diagnosis of the tumour returned as a borderline phyllodes tumour with moderate pleomorphism and three to four mitoses per ten high power fields (Figure 4). Areas of myxoid degeneration were present without any lymphovascular invasion.

Four months later she returned with a rapidly progressing local recurrence of phyllodes tumours in the same quadrants of the breast for which she underwent a simple mastectomy.

## Discussion

**Figure 5:**
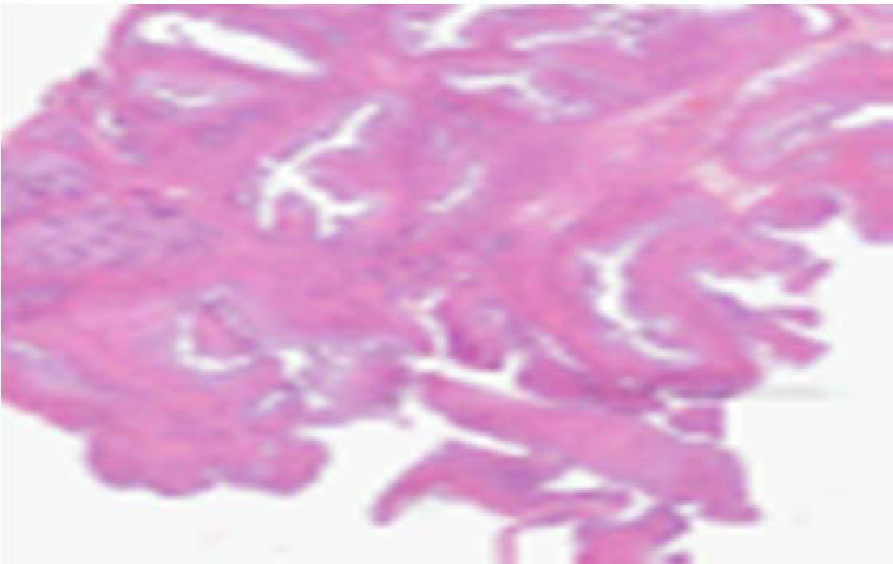
Benign Phyllodes tumour histology (haematoxylin and eosin stained) showing benign epithelia and a spindle stoma.

Phyllodes tumours of breast are rare (2.1 per million) and usually benign. They are more common in Latin American (Hispanic) women [7]. On histology they are fibro-epithelial tumours similar to fibroadenomas except for the hypocellular stroma with few mitoses and a true capsule present in fibroadenoma [3]. They occur over a wide age range with a median age of 45 years, 20 years later than fibroadenoma. It has been postulated that stromal induction of phyllodes tumour can occur as a result of growth factor produced by the breast epithelium [9]. The presence of sex hormones in pregnancy may stimulate the growth of these tumours. Triple assessment by clinical, radiological and cytological or histopathological examination individually or in combination gives poor diagnostic accuracy for phyllodes tumours [1,10]. Macroscopically, small tumours usually resemble fibroadenomas but tumours can grow rapidly to over 20 cm in diameter [2]. Involvement of axillary nodes is rare in phyllodes tumour and axillary dissection is not indicated. However, axillary lymph node metastases occur in about 10% of patients with malignant phyllodes [2]. The two small impalpable axillary lymph nodes identified by ultrasound were probably reactive. Needle core biopsy rarely produces a definite pre-operative diagnosis because this tumour shares many cytological features with fibroadenoma. One series showed a pre-operative diagnosis of phyllodes tumour in only 10% to 20% of cases [11]. Large tumours often have a fleshy appearance with cystic and necrotic areas (Figure 5) [2]. Microscopically, epithelioid-lined cystic spaces with hypercellular stroma confirms the diagnosis. An equivocal diagnosis of phyllodes tumour should not prevent excision if clinical suspicion remains [12].

Wide tumourectomy is the mainstay of treatment and the high recurrence rate of 37% in one series justifies wide margin excision [13]. Local recurrence rates are correlated with positive excision margins. [11] Although mastectomy has invariably been performed for larger tumours, to date, there is no correlation between tumour size and risk of local recurrence. Other studies have shown a higher risk of local recurrence in borderline and malignant tumours [1,5]. By contrast patients treated with mastectomy (subcutaneous, simple, modified-radical) showed no evidence of local recurrence [14]. Local recurrence usually occurs within the first few years of surgery and histologically resembles the original tumour [12]. The recurrences in our patient may have been due to incomplete excisions as recurrence occurred in the same quadrants of the breast. Thus intra-operative ultrasound may be more useful than palpation in detecting the phyllodes tumours. Patients with giant phyllodes may have clinically enlarged axillary lymph nodes that may be suspicious for metastatic disease. The surgeon may be forced to proceed with axillary lymph node dissection especially as sentinel lymph node biopsy may not be accurate in these patients [5]. The controversy between breast-conserving surgery and mastectomy remains because of the unpredictable nature of the disease. Contraindications for breast- conserving surgery include large tumours, multifocality, borderline histology and malignancy because of their greater risk of recurrence. However, local recurrence can always be managed by further wide excision if there is no evidence of borderline pathology or malignancy [1,2,12]. Histological type is the most important predictor for metastatic spread, although it may not correlate with clinical behaviour because both malignant and borderline tumours are capable of metastasizing. The five year survival for benign, borderline or malignant tumours is 96%, 74% and 66% respectively [15]. Tumour size is an important factor in predicting metastatic spread. This may correlate with the necrotic elements in large tumours and the fact that tumour necrosis is a histological prognostic factor albeit of a small effect on its own [16]. The risk of incomplete excision and the size of the tumour in relation to the breast is an onco-plastic reason why mastectomy should be performed for large tumours. A clear surgical margin is the only proven protective factor [1,2].

There is no contraindication to immediate reconstruction after mastectomy in cases of giant phyllodes tumours [17]. Malignant phyllodes tumours are best managed with wide excision of normal breast tissue around the tumour to obtain a 1 cm margin of normal- appearing breast tissue, but very large malignant phyllodes tumours require mastectomy [6]. With malignant tumours, 22% may give rise to haematogenous metastasis most frequently to the lungs. The role of adjuvant therapies (radiotherapy and chemotherapy) is presently undefined and should be tested in multicentre, prospective, randomized trials [18].

## Conclusions

This case highlights the arguments for and against the treatment of phyllodes tumour of the breast by breast-conserving surgery. Breast-conserving surgery (wide tumourectomy) may suffice for phyllodes tumour as it has the advantage of cosmesis in a relatively benign disease. It is not ideal for giant, multifocal phyllodes and phyllodes of borderline or malignant pathology. Close follow-up is required for detecting and treating local recurrence following breast- conserving surgery.

## Consent

Written informed consent was obtained from the patient for publication of this case report. A copy of this written consent is available for review by the Editor-in-Chief of this journal.

## Competing interests

The authors declare that they have no competing interests.

## Authors’ contribution

EPW is the main author and surgeon. GEO is the pathologist. MNN contributed to the review of manuscript. LA carried out the literature search. All authors read and approved the final manuscript.
